# The Genomic Landscape of Romanian Non-Small Cell Lung Cancer Patients: The Insights from Routine NGS Testing with the Oncomine^TM^ Dx Express Test at the PATHOS Molecular Pathology Laboratory

**DOI:** 10.3390/cancers17121947

**Published:** 2025-06-11

**Authors:** Orsolya I. Gaal, Andrei Ungureanu, Bogdan Pop, Andreea Tomescu, Andreea Cătană, Milena Man, Ruxandra Mioara Râjnoveanu, Emanuel Palade, Marioara Simon, Stefan Dan Luchian, Milan Paul Kubelac, Annamaria Fulop, Zsolt Fekete, Tudor Eliade Ciuleanu, Ion Jentimir, Bogdan Popovici, Calin Cainap, Alexandra Cristina Preda, Cosmina Magdau, Andrei Lesan, Bogdan Fetica

**Affiliations:** 1 Department of Molecular Sciences, Medical Genetics, “Iuliu Hatieganu” University of Medicine and Pharmacy, 400006 Cluj-Napoca, Romania; ildiko.orsolyagaal@radboudumc.nl (O.I.G.); catanaandreea@gmail.com (A.C.); 2 Department of Genetic Explorations, “Prof. Dr. Ion Chiricuta” Institute of Oncology, 400015 Cluj-Napoca, Romania; 3 Molecular Pathology Laboratory Pathos, 400394 Cluj-Napoca, Romania; andreeatomescu98@yahoo.com (A.T.); feticab@yahoo.com (B.F.); 4 Amethyst Medical Center, 407280 Cluj-Napoca, Romania; andreiungureanu.oncolog@yahoo.com; 5 Department of Morphological Sciences, Pathology, “Iuliu Hatieganu” University of Medicine and Pharmacy, 400006 Cluj-Napoca, Romania; 6 Department of Pathology, “Prof. Dr. Ion Chiricuta” Institute of Oncology, 400015 Cluj-Napoca, Romania; fannam83@yahoo.com; 7 Department of Medical Specialties, Pulmonology, “Iuliu Hatieganu” University of Medicine and Pharmacy, 400006 Cluj-Napoca, Romania; manmilena50@yahoo.com (M.M.); andrei_lesan@yahoo.com (A.L.); 8 “Leon Daniello” Clinical Hospital for Pneumophthisiology, 400332 Cluj-Napoca, Romania; ruxandra.rajnoveanu@umfcluj.ro (R.M.R.); paladeemanuel1@gmail.com (E.P.); simonmariaro@gmail.com (M.S.); dr.ijentimir@gmail.com (I.J.); cmagdau@yahoo.com (C.M.); 9 Department of Oncology, Palliative Medicine, “Iuliu Hatieganu” University of Medicine and Pharmacy, 400006 Cluj-Napoca, Romania; 10 Department of Surgery, Vascular, Cardiovascular and Thoracic Surgery, “Iuliu Hatieganu” University of Medicine and Pharmacy, 400006 Cluj-Napoca, Romania; 11 Department of Oncology, County Hospital Satu Mare, 440030 Satu Mare, Romania; luchiands@yahoo.com; 12 Star Medica Clinic, 447230 Satu Mare, Romania; 13 Department of Oncology, Medical Oncology, “Iuliu Hatieganu” University of Medicine and Pharmacy, 400006 Cluj-Napoca, Romania; cabinetkubelac@gmail.com (M.P.K.); ctecabinet@gmail.com (T.E.C.); cabinetcainap@gmail.com (C.C.); preda.alexandra_cristina@yahoo.com (A.C.P.); 14 Department of Medical Oncology, “Prof. Dr. Ion Chiricuta” Institute of Oncology, 400015 Cluj-Napoca, Romania; 15 STAR Institute, Babeș-Bolyai University, 1 Mihail Kogălniceanu Street, 400347 Cluj-Napoca, Romania; 16 Department of Oncology, Oncology Radiotherapy, “Iuliu Hatieganu” University of Medicine and Pharmacy, 400006 Cluj-Napoca, Romania; drfekete@yahoo.com; 17 Department of Radiotherapy, “Prof. Dr. Ion Chiricuta” Institute of Oncology, 400015 Cluj-Napoca, Romania; 18 Department of Surgical Oncology, “Prof. Dr. Ion Chiricuta” Institute of Oncology, 400015 Cluj-Napoca, Romania; bpopovici10@yahoo.com

**Keywords:** lung cancer, NSCLC, adenocarcinoma, NGS, precision oncology

## Abstract

**Background:** Comprehensive molecular profiling is essential for precision oncology in non-small cell lung cancer (NSCLC). However, genomic data from Eastern European populations, including Romania, remain limited. **Methods:** We analyzed 398 consecutive NSCLC cases tested at the PATHOS Molecular Pathology Laboratory (Cluj-Napoca, Romania) between April 2024 and February 2025 using the Ion Torrent™ Genexus™ System and the Oncomine™ Dx Express Test, which evaluates SNVs/indels in 46 genes, fusions in 23 genes, and CNVs in 19 genes from FFPE samples. **Results**: The cohort was predominantly male (66%) with a median age of 67 years. Adenocarcinoma represented 70% of cases with known histology. Genomic profiling revealed a high frequency of actionable alterations. *KRAS* mutations were the most common (29.1%), with p.G12C detected in 10.3% of all the cases. *EGFR* mutations were present in 14.3% of patients, mostly exon 19 deletions and L858R substitutions. *BRAF* alterations (5.3%) included both V600E and non-V600E variants. RET alterations were detected as eight missense mutations, two canonical fusions (*KIF5B*–*RET*, *CCDC6*–*RET*), one amplification, and three transcript imbalances. *EML4-ALK* fusions (1.77%), *ERBB2* mutations/amplifications (3.0%), and *FGFR1/FGFR3* amplifications were also observed. **Conclusions**: This study provides the first large-scale molecular snapshot of NSCLC in Romania. While the overall genomic profiles align with Western populations, the higher frequency of KRAS p.G12C and *FGFR* amplifications highlights the value of region-specific data to support targeted therapies in Eastern Europe.

## Simple Summary

This study looked at the genetic makeup of lung cancer in Romanian patients to help guide more personalized treatments. Researchers analyzed 398 cases of non-small cell lung cancer (NSCLC) using advanced technology that detects mutations in cancer-related genes. Most patients were older men, and the most common tumor type was adenocarcinoma. The most frequent mutation found was in the *KRAS* gene (especially the p.G12C variant), followed by changes in *EGFR* and *BRAF* genes. Less common but important alterations were found in *RET*, *ALK*, *ERBB2*, and *FGFR* genes. These genetic changes can be targeted with specific treatments, which means patients could benefit from drugs designed for their unique cancer profile. This is the first large-scale study of its kind in Romania and shows that local data is essential for guiding cancer therapy, as some gene changes were more common in Romanian patients compared to other populations.

## 1. Introduction

Lung cancer continues to represent a major contributor to global cancer-related mortality, with non-small cell lung cancer (NSCLC) comprising approximately 85% of all diagnosed cases [1]. Despite advances in early detection and treatment, the prognosis for lung cancer patients remains poor, largely due to late-stage diagnosis and the molecular heterogeneity of the disease [2,3]. In recent years, the integration of molecular profiling into clinical practice—particularly through next-generation sequencing (NGS)—has revolutionized the diagnostic and therapeutic landscape of lung cancer by enabling personalized treatment strategies based on actionable genetic alterations. Therefore, in the context of NSCLC, an “actionable” mutation refers to a specific genetic alteration within tumor DNA that can be targeted by an existing or investigational therapy, leading to a clinically meaningful response. These mutations are identified through molecular diagnostic testing and are associated with predictive value, as they predict sensitivity or a resistance to specific targeted therapies. Also, there are available drugs (approved or in clinical trials) that specifically inhibit the aberrant protein function resulting from the mutation [4]. Targeting these mutations has been shown to improve clinical outcomes such as progression-free survival, overall survival, and quality of life. Identifying actionable mutations enables personalized treatment strategies, allowing clinicians to select the therapies that are more likely to be effective based on the tumor’s genetic profile. This approach is a cornerstone of precision oncology, aiming to optimize the treatment efficacy while minimizing unnecessary toxicity.

The therapeutic landscape of NSCLC has been transformed by the discovery of recurrent, targetable genomic alterations—most commonly involving *EGFR*, *ALK*, *ROS1*, *BRAF*, *MET*, *RET*, *KRAS*, and *NTRK* genes—together with the emergence of immune-checkpoint inhibitors guided by PD-L1 expression and tumor mutational burden (TMB) [5,6,7]. Consequently, comprehensive molecular profiling at the diagnosis has become an essential step in clinical decision-making, as highlighted by current international guidelines (NCCN, ESMO) [8,9].

Although the prevalence and spectrum of actionable mutations have been extensively characterized in large international cohorts [10], European population-specific data remain heterogeneous, and information from Eastern Europe—particularly Romania—is scarce [11]. Romania bears a high burden of lung cancer, with the age-standardized incidence (world standard) estimated at 40.4 per 100,000 and a persistently high smoking prevalence, yet nationwide access to next-generation sequencing (NGS) has only recently become available [12]. Comprehensive profiling initiatives, such as those conducted by The Cancer Genome Atlas (TCGA), East Asian consortia, and pan-European registries, have contributed substantially to mapping the global distribution of driver mutations; however, representation from Eastern Europe remains limited [13]. *EGFR* mutations, for example, are significantly more common in East Asian populations—affecting 30–50% of adenocarcinomas—compared to Western populations, where the prevalence ranges from 10–15% in North America and Europe [14,15]. In contrast, *KRAS* mutations are predominant in Western cohorts, identified in approximately 25–30% of NSCLC cases, whereas they occur less frequently in Asian populations (5–15%) [16,17,18]. Similarly, *ALK* rearrangements are observed in 3–7% of NSCLC globally, with somewhat higher frequencies in Asian never-smoker cohorts [19,20,21]. These epidemiological differences highlight the importance of generating population-specific genomic data to optimize biomarker-guided therapies and address the regional disparities in the access to targeted treatments. Therefore, generating local real-world evidence is critical for tailoring precision-oncology strategies to the Romanian population and for identifying potential disparities in the biomarker distribution, the turnaround time, and the therapy allocation.

In consequence, all the mutations reported in this study were detected using the Oncomine^TM^ Dx Express Test on the Ion Torrent Genexus System, which enables the detection of SNVs, indels, fusions, and CNVs from FFPE-derived DNA/RNA. The panel is based on Ion AmpliSeq technology, with variant interpretation supported by the Oncomine Knowledge Reporter, a curated clinical database aligned with evidence levels from regulatory approvals and clinical trials. The panel used in our study is designed to detect somatic mutations, and testing was exclusively performed on tumor tissue. Nevertheless, the mutations we report (e.g., *EGFR* p.L858R, *KRAS* p.G12C, *BRAF* V600E, and *RET* fusions) are well-established as oncogenic drivers with virtually no physiological role in normal adult lung tissue and are not typically found in nonmalignant tissue unless in precancerous or metaplastic conditions.

In this context, we report the molecular profile of a cohort of Romanian patients diagnosed with NSCLC who were evaluated using NGS at the PATHOS Molecular Pathology Laboratory in Cluj-Napoca. In addition, this present study reports the clinicopathological features and mutational landscape of a consecutive cohort of Romanian patients with histologically confirmed NSCLC who underwent routine NGS testing with the Oncomine Dx panel on the Genexus platform at the PATHOS Molecular Pathology Laboratory. Therefore, by providing the first comprehensive genomic snapshot from a Romanian tertiary center, we aim to inform national precision-oncology initiatives and contribute to the wider European database on lung-cancer molecular epidemiology.

## 2. Materials and Methods

### 2.1. Eligibility

We included in this study patients diagnosed with non-small cell lung cancer (NSCLC), either in our laboratory or in external facilities, based on histopathological evaluation, complemented by immunohistochemistry when necessary. For cases diagnosed externally, the information regarding the cancer type was obtained from the accompanying test request form, which confirmed at a minimum a diagnosis of NSCLC, although subtyping was not mandatory. The cohort comprises both newly diagnosed (treatment-naïve) and previously treated patients who underwent testing for 46 genes using the Oncomine^TM^ Dx Express Test Multi-CDx System between April 2024 and February 2025 in our laboratory.

### 2.2. Study Design

In this present study, formalin-fixed, paraffin-embedded (FFPE) tissue sections (4–5 µm thick) or pleural effusion cell pellets from biopsy samples were submitted to the PATHOS Molecular Pathology Laboratory for molecular analysis. Nucleic acid extraction (both DNA and RNA) was performed using the Genexus™ Purification System (Thermo Fisher Scientific, Waltham, MA, USA), ensuring standardized and reproducible processing. The laboratory implemented a next-generation sequencing (NGS) workflow based on the Oncomine™ Dx Express Test (Thermo Fisher Scientific, Waltham, MA, USA), integrated with the Ion Torrent™ Genexus™ System. This CE-IVD–marked platform enables same-day library preparation, sequencing, and automated data analysis, delivering results within 72 h [22]. The assay interrogates 46 genes for single nucleotide variants (SNVs) and short insertions/deletions (indels), 19 genes for copy number alterations, and 23 genes for gene fusions, providing a comprehensive, guideline-concordant molecular panel [23,24]. Variant annotation and interpretation were conducted using the Oncomine™ Knowledge Reporter version 5.5.1, a curated clinical decision support tool. Moreover, when the sequencing data is paired with the Oncomine Precision Assay, the platform has shown performance that matches or exceeds that of other next-generation sequencing methods in detecting DNA and RNA variants from both FFPE tissue and liquid-biopsy specimens across multiple tumor types [24]. Additional clinical data included the patient sex, the age at the specimen collection, the disease stage, the histological subtype, the PD-L1 expression status, and the smoking history.

## 3. Results

Between April 2024 and February 2025, the PATHOS Molecular Pathology Laboratory received 398 consecutive NSCLC specimens for routine genomic profiling with the Ion Torrent Genexus + Oncomine^TM^ Dx Express Test. The demographic characteristics of the 398 patients whose samples underwent analysis with the Oncomine^TM^ Dx Express Test (ODxET) are summarized in Table 1. The cohort was predominantly male, comprising approximately 66% of cases, with a male-to-female ratio of 1.99:1. The mean age at diagnosis was 65.94 years, with a median of 67 years. No statistically significant difference in the age was observed between the male and female patients. The majority of individuals (approximately 67%, *n* = 267) were diagnosed in their sixth or seventh decade of life, while 90% (*n* = 353) were diagnosed between the ages of 50 and 79 years. A smaller proportion (*n* = 20) were aged over 80 years, and the remaining cases were between 38 and 50 years of age at diagnosis.

Out of the total study population, the histopathological subtype data for non-small cell lung cancer (NSCLC) was available for 294 cases, representing approximately 74% of the cohort. Among these, the predominant subtype was adenocarcinoma, accounting for approximately 70% (*n* = 207) of the cases with available data. This was followed by NSCLC not otherwise specified (NOS) at approximately 15% (*n* = 44) and squamous cell carcinoma, representing approximately 11% (*n* = 33). Large-cell neuroendocrine carcinoma (LCNEC) and adenosquamous carcinoma collectively accounted for the remaining 3.4% (*n* = 10) of cases.

**Figure 1:**
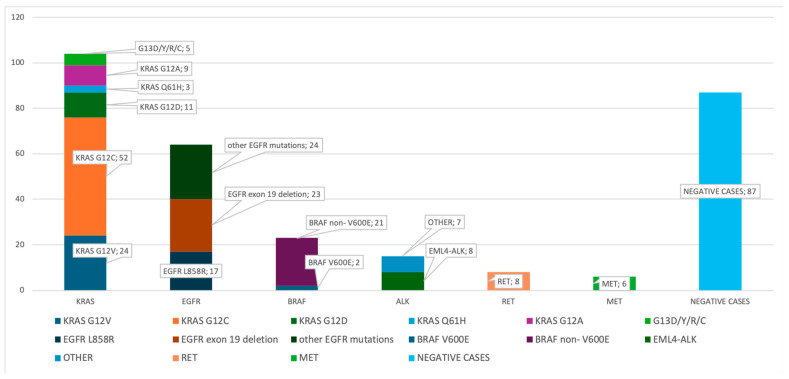
The distribution of the driver mutations detected using ODxET. The distribution of the driver gene mutations detected using ODxET at the PATHOS Molecular Laboratory is shown in the bar chart (*n* = 398).

The molecular analysis focused on three categories of genomic alterations: single-nucleotide variants (SNVs)/indels, gene fusions, and copy number variations (CNVs). A wide range of somatic DNA alterations was detected. The spectrum was dominated by missense substitutions (92%), with a minority of nonsense (2.5%) and frameshift events. The most frequently altered genes included *KRAS* (*n* = 116), *EGFR* (*n* = 57), *BRAF*, *TP53*, *ERBB2*, and *PIK3CA*, each identified in 5–7% of tumors. Overall, 259 patients (64.9%) carried at least one molecular alteration for which an EMA- or FDA-approved therapy or an active phase II/III clinical trial is available (Figure 1) (Table 2). The actionable alteration landscape included the following: *KRAS* mutations were identified in 116 samples (29.1%), with codon 12 substitutions (notably p.G12C, p.G12V, and p.G12D) accounting for over 85% of cases. Within the adenocarcinoma subset, the *KRAS* mutation rate was 32.4%. Among these, the p.G12C was the most frequently mutated variant, detected in 52 samples. *BRAF* mutations were identified in 20 samples (5.3%), with a wide spectrum of activating variants. The most common were p.G469 substitutions, accounting for nearly half of the cases (G469V, G469E, and G469A). *BRAF* p.V600E, a well-established targetable driver mutation, was detected in two tumors, while other pathogenic variants included K601N/E, D594G, N581S, and L597R. These mutations were distributed across adenocarcinoma and NSCLC-NOS subtypes and were predominantly of the missense type, suggesting their potential relevance for targeted therapeutic intervention.

EGFR mutations were present in 57 cases (14.3%), most commonly as exon 19 deletions (61.4%) and exon 21 p.L858R substitutions (31.6%). One treatment-naïve case harbored primary p.T790M mutations. *TP53* was the most frequently mutated gene, found in 143 cases (35.9%), with the most mutations affecting the DNA-binding domain. Further, gene fusion analysis revealed several clinically relevant alterations. The most commonly identified fusions involved *ALK*, *RET*, *FGFR3*, and *EML4-NTRK3*. Notably, *ALK* rearrangements were the most common (26.47% of fusion-positive cases), with seven confirmed *EML4-ALK* variants. Additional recurrent fusions involved *RET*, *FGFR3-TACC3*, and *MET* exon 14 skipping. Nevertheless, fusion-positive cases were predominantly observed in younger patients (<70 years), most often with adenocarcinoma or Pulmonary large-cell neuroendocrine carcinoma (LCNEC) histology.

In addition, *RET* mutations were identified in eight samples (2.0%), all of which were missense substitutions affecting the tyrosine kinase domain. The most frequently observed variant was c.2372A>T, present in four cases. Other pathogenic alterations included p.C620F and p.R912W, both of which have been previously associated with oncogenic signaling in RET-driven cancers. These mutations were primarily detected in adenocarcinoma samples, underscoring their relevance for targeted RET inhibition in this subgroup. In terms of copy number alterations, one case showed *RET* amplification, with a copy number value of 9.79, supporting high-level gene amplification. Moreover, the RNA fusion analysis revealed two gene fusions *KIF5B*–*RET* and *CCDC6*–*RET*. Three additional cases exhibited *RET* imbalance, a signal consistent with potential gene rearrangement but requiring further confirmatory testing. Furthermore, *ROS1* alterations were not identified in this cohort through either DNA variant analysis or RNA-based fusion detection. This suggests a low prevalence of ROS1-driven oncogenic events in this Romanian NSCLC population.

## 4. Discussions

This study provides one of the earliest in-depth molecular characterizations of non-small cell lung cancer (NSCLC) in Romanian patients, utilizing next-generation sequencing (NGS) performed with the Ion Torrent™ Genexus™ System in combination with the Oncomine™ Dx Express Test. The analysis revealed a high prevalence of clinically actionable mutations, including alterations in *KRAS*, *EGFR*, *TP53*, and *ALK*, alongside notable incidences of *FGFR1/FGFR3* amplifications. These findings not only align with genomic profiles reported in other European [18,25] and North American cohorts [16,26] but also highlight distinct regional patterns that may reflect underlying genetic, environmental, or epidemiological differences. Moreover, epidemiological studies have demonstrated that the prevalence of epidermal growth factor receptor (*EGFR*) mutations in non-small cell lung cancer (NSCLC) varies significantly across geographic regions. In North American populations, *EGFR* mutations are observed in approximately 10–15% of NSCLC cases [27,28]. In contrast, Asian cohorts exhibit a markedly higher prevalence, with frequencies ranging from 30% to 50%, and reaching up to 57% in some East Asian countries, such as Taiwan [14]. These interregional differences are believed to arise from a complex interplay of factors, including the ethnic genetic predisposition, environmental exposures, and variations in tobacco use patterns. Such findings highlight the critical need for region-specific molecular profiling to inform the selection of targeted therapies and optimize precision oncology approaches for NSCLC patients. Conversely, *KRAS* mutations were identified in 29.1% of cases in the Romanian cohort, with the G12C variant accounting for 10.3%. These findings are consistent with North American data, where *KRAS* alterations are observed in 25–30% of NSCLC cases and the G12C variant occurs in approximately 13% [29,30]. In contrast, *KRAS* mutations are significantly less prevalent in Asian populations, where frequencies range from 5% to 12%, further supporting the notion that distinct tumorigenic mechanisms may be at play across the populations [31,32].

*BRAF* mutations were detected in 21 cases (5.3%), with a heterogeneous mutation profile. Although p.V600E—the most clinically actionable *BRAF* variant—was present in only two cases, the majority of alterations affected the codons G469, K601, and D594, which are increasingly recognized as oncogenic. This prevalence aligns with the rates reported in American populations (3.5–4%) [33,34], where p.V600E predominates, but differs from Asian cohorts, where *BRAF* mutation frequencies are typically lower (~1–2%) and often non-V600E in nature [35,36]. These findings suggest that, in Romania, *BRAF*-mutant NSCLC is not rare and may require broader molecular interrogation beyond p.V600E hotspots.

*RET* alterations were identified through both DNA and RNA analyses. A total of eight missense *RET* mutations were detected in the DNA panel, including oncogenic variants such as p.C620F and p.R912W. In the RNA fusion module, two canonical *RET* fusions (*KIF5B*–*RET* and *CCDC6*–*RET*) were confirmed, while three additional samples exhibited *RET* transcript imbalance, suggesting possible rearrangements. Additionally, one case exhibited *RET* amplification with a high copy number (>9). The overall frequency of *RET* fusions in our cohort (~1.3%) is consistent with the reported 1–2% prevalence in NSCLC/ADC patients of both Asian and European ancestry [37]. Multiple studies suggest that *RET* fusions are more commonly found in younger individuals who are never-smokers or light smokers [38,39]. Moreover, the most frequently observed *RET* fusion partners in NSCLC are *KIF5B* and *CCDC6*. These fusions lead to constitutive activation of the *RET* tyrosine kinase, promoting oncogenic signaling [40].

Regarding *ROS1*, neither SNVs nor RNA-detectable gene fusions were identified in our cohort. This is consistent with the relatively low prevalence of ROS1 rearrangements globally, typically ranging from 1–2% in unselected NSCLC cohorts [41].

Furthermore, *ALK* rearrangements, primarily involving *EML4-ALK* fusions, were present in 1.77% of cases. Globally its prevalence is estimated at approximately 3–7% in unselected NSCLC populations [42]. This fusion is more commonly observed in specific subgroups, such as younger patients, non-smokers, and those with adenocarcinoma histology. For instance, a meta-analysis encompassing various populations reported an overall incidence rate of 6.8% for *EML4–ALK* fusions in NSCLC patients [20]. Notably, certain Asian populations, including those in China, have reported higher frequencies of *ALK* rearrangements [19,21,43], potentially due to inherited genetic variants or population-specific environmental risk factors. Moreover, alterations in *ERBB2* (*HER2*), including mutations and gene amplifications, were observed in 3.0% of cases, consistent with international data indicating *ERBB2* alteration rates between 2% and 4% [44]. Given their established role as actionable targets, these findings underscore the importance of including ERBB2 in routine molecular testing panels for NSCLC.

Finally, a relatively high incidence of *FGFR1* and *FGFR3* amplifications was noted in this cohort (12 and 11 cases, respectively). While FGFR alterations are less frequently reported in global NSCLC datasets, their enrichment in this Romanian population suggests the possibility of region-specific oncogenic drivers that merit further molecular and clinical investigation. However, an important consideration in interpreting our results is the distribution of histological subtypes within the study cohort, in which adenocarcinoma accounted for approximately 70% of cases. This proportion is notably higher than that reported in national epidemiological data from Romania. According to a retrospective analysis of lung cancer cases diagnosed between 2001 and 2010, squamous cell carcinoma (SCC) was the predominant subtype, representing approximately 47% of cases, while adenocarcinoma accounted for only 38% [45]. This distribution contrasts with global trends, where adenocarcinoma has become the most frequent histological subtype, particularly in high-income countries. The higher prevalence of SCC in Romania may be attributed to elevated smoking rates—particularly among men—and the lack of a national lung cancer screening program, both of which contribute to late-stage detection and may skew the histological landscape.

A major strength of this study is the use of both DNA and RNA-based analyses, enabling simultaneous detection of a broad spectrum of genomic alterations, including single-nucleotide variants, short indels, gene fusions, and copy number variations, in a clinically actionable timeframe. Additionally, this study provides region-specific genomic data for NSCLC, addressing a notable gap in the Eastern European molecular oncology literature. The relatively large, consecutive case series enhances the representativeness of the findings for Romanian patients, while the identification of rare but actionable mutations (e.g., *MET* exon 14 skipping, *RET* rearrangements, and *FGFR* amplifications) supports the utility of comprehensive profiling beyond standard targets. However, this study also has several limitations. Firstly, it is a single-center, retrospective analysis, which may introduce selection bias and limit the generalizability of the findings. Secondly, the clinical outcome data, including the treatment responses and the survival metrics, were not available for correlation with molecular alterations. Thirdly, while the panel covers key guideline-recommended biomarkers, it does not assess the tumor mutational burden (TMB) nor microsatellite instability (MSI), which are increasingly relevant for immunotherapy stratification. Nonetheless, a potential limitation of this study is the overrepresentation of adenocarcinoma cases, which may not accurately reflect the true national distribution of lung cancer histological subtypes in Romania. This imbalance likely stems from referral and testing practices at our tertiary care center, where molecular profiling is more frequently pursued for non-squamous NSCLC, consistent with international recommendations (e.g., NCCN, ESMO) that prioritize testing for actionable mutations primarily in adenocarcinomas. Additionally, squamous cell carcinoma cases may have been underrepresented due to diagnostic limitations such as insufficient tumor material or suboptimal sample quality, as well as a lower anticipated yield of targetable alterations, potentially leading to selective submission for NGS analysis.

Therefore, future studies incorporating multi-institutional cohorts, prospective clinical data, and expanded genomic profiling (including TMB, MSI, and the methylation status) would be valuable to validate and extend these findings. Furthermore, RNA degradation affected 28 samples, preventing successful gene fusion and transcript-level analyses in these cases, thereby potentially underestimating the true prevalence of actionable fusions. Nevertheless, it is worth mentioning that somatic alterations identified in this study were exclusively derived from formalin-fixed, paraffin-embedded tumor tissue, and no matched normal controls were available to empirically validate the somatic origin. However, the panel utilized—Oncomine^TM^ Dx Express Test—targets well-characterized oncogenic driver mutations with established roles in tumorigenesis and minimal physiological relevance in non-neoplastic tissues. Notably, mutations such as *EGFR* exon 19 deletions and p.L858R, *KRAS* codon 12 substitutions (e.g., p.G12C and p.G12V), *BRAF* p.V600E, and canonical *RET* fusions (*KIF5B–RET*, *CCDC6–RET*) are exceedingly rare in normal somatic tissues and are considered hallmark somatic events in non-small cell lung cancer. Importantly, mutations in *TP53* or components of the RAS pathway have occasionally been observed in histologically normal bronchial epithelium or premalignant lesions, although such occurrences are infrequent and often subclonal [46,47]. Future directions should include larger, multi-institutional cohorts, integration of clinical outcomes, and expanded genomic interrogation including TMB, MSI, and epigenetic features, to further refine biomarker-driven therapy in NSCLC within this population.

## 5. Conclusions

In conclusion, the integration of the Oncomine™ Dx panel with the Genexus™ platform enabled rapid and comprehensive molecular profiling in a real-world clinical setting. The genomic landscape of this exclusively Romanian NSCLC cohort was broadly consistent with findings from larger European studies but showed a relatively high prevalence of *KRAS* p.G12C mutations and a notable incidence of *FGFR1/FGFR3* amplifications. These results highlight the importance of region-specific molecular data to inform precision oncology strategies and optimize patient stratification for targeted therapies in Eastern Europe.

## Figures and Tables

**Table 1:** Clinical background of patients who underwent ODxET testing.

Characteristics	Number (%)
Age	
median (range)	67.5 (38–94)
Sex	
Male	265 (66.58)
Female	133 (33.41)
Histology	
Adenocarcinoma	207 (70.41)
Squamous Cell Carcinoma	33 (11.2)
NSCLC, NOS	44 (14.97)
LCNEC	7 (2.38)
Adenosquamous Carcinoma	3 (1.02)
cases without details regarding diagnosis	104 (26.13)

Abbreviation: ODxET, Oncomine^TM^ Dx Express Test.

**Table 2:** Prognostic outcomes by actionable NSCLC driver mutations.

Mutation (Target)	Median Overall Survival (mOS)	Median Progression-Free Survival (mPFS)	Key Findings and Notes	Therapy	Trial (Phase)
EGFR	36.9–40.6 months	11.2–14.2 months	Third-generation EGFR-TKIs (e.g., osimertinib) improve survival; uncommon mutations may have shorter PFS.	Osimertinib	FLAURA (Phase III)
KRAS	12.6–16.1 months	4.0–5.1 months	KRAS mutations associated with poorer prognosis; G12C subtype may have slightly better outcomes.	Sotorasib	CodeBreaK 100 (Phase II)
ALK	Not reached	18.5–43.2 months	With longer follow-up, brigatinib showed efficacy and tolerability versus crizotinib in patients with or without poor prognostic biomarkers	Brigatinib	ALTA-1L Trial
ALK	55.4 months	15.0–34.8 months	ALK inhibitors (e.g., alectinib) significantly extend survival; resistance remains a challenge.	Alectinib	ALEX (Phase III)
BRAF	13.0–19.0 months	5.0–7.0 months	BRAF mutations may respond to targeted therapies; V600E subtype shows better outcomes.	Dabrafenib + Trametinib	BRF113928 (Phase II)
MET exon 14	10.7–19.0 months	6.6–7.3 months	MET inhibitors (e.g., capmatinib) improve outcomes; concurrent mutations may affect efficacy.	Capmatinib	GEOMETRY mono-1 (Phase II)
RET	17.5–34.3 months	11.8 months	Selective RET inhibitors (e.g., pralsetinib) enhance survival; prior treatments may impact efficacy.	Pralsetinib	ARROW (Phase I/II)
ROS1	47.8–52.1 months	15.7–19.3 months	ROS1 TKIs (e.g., crizotinib, entrectinib) yield favorable outcomes; fusion partner may influence response.	Crizotinib	PROFILE 1001 (Phase I)
ROS1	Not evaluable 25.1 months (in ROS1 TKI-pretreated and chemotherapy-naïve cohort)	35.7 months	High rates of response and durable response in ROS1 fusion–positive NSCLC after treatment with Repotrectinib	Repotrectinib	TRIDENT-1 (Phase II)
ERBB2 (HER2)	17.7–25.8 months	4.4 months	HER2 mutations associated with variable outcomes; targeted therapies under investigation.	Trastuzumab Deruxtecan	DESTINY-Lung01 (Phase II)

## Data Availability

The datasets used and/or analysed during the current study are available from the corresponding author on reasonable request.
